# Evolutionary game analysis between employees and employers about working overtime from the perspective of information asymmetry

**DOI:** 10.1186/s40359-022-00802-y

**Published:** 2022-04-09

**Authors:** Junjie Dong, Shumin Yan

**Affiliations:** School of Economics and Management, Tongji University, Shanghai, China

**Keywords:** Evolutionary game, Voluntary overtime, Involuntary overtime, Overtime pay, Information asymmetry, Principal-agent theory

## Abstract

**Background:**

Overtime is an international phenomenon, especially in some Chinese Internet technology companies, the 996 work regime is a common corporate atmosphere. This paper holds that overtime work is the result of a long-term dynamic game between employees and employers. In such a dynamic evolution process, employers and employees both cooperate and conflict, they will choose a strategy conducive to their own development through long-term learning and improvement.

**Methods:**

Based on the evolutionary game theory and principal-agent theory, this paper constructs a $2\times 2$ evolutionary game matrix. The strategies of employees can be divided into voluntary overtime and involuntary overtime, while the strategies of employers can be divided into providing overtime pay and not providing overtime pay. The stability of the system is related to four parameters: resource consumption, information asymmetry coefficient, trust coefficient, and moral hazard coefficient.

**Results:**

Through an in-depth study of the model and data simulation, the system has five equilibrium points, an ESS point, and a saddle point in any case. Accordingly, we put forward two theorems and three propositions, which are verified not only theoretically but also by data simulation. Besides, the strategies of the employees and the employers will evolve from the initial state to (Involuntarily, Not pay) or (Voluntarily, Pay) under different situations. This is closely related to the initial parameters of the evolutionary game model and the payment matrix.

**Conclusions:**

By summarizing the influence of each parameter on the evolutionary path, we believe that fairness and information equivalence between employees and employers can effectively promote both parties to reach the Pareto optimal state. In other words, employees and employers need to communicate and share information promptly to ensure the unity of information acquired by each other and achieve a win–win situation. This paper contributes to providing theoretical guidance and practical enlightenment for organizations to manage employees' overtime behavior scientifically and improve their work psychology reasonably.

## Background

Working hours are getting longer all over the world, and overtime has become very common, especially in China. Nowadays, China not only leads the world in economic growth but also attracts attention from other countries for its long-term and stable development. However, the development of productivity and the improvement of economic level have not freed employees from heavy work. On the contrary, overtime work has gradually become a new normal and a new culture in the workplace. According to the disclosure of the National Bureau of Statistics in April 2021, the average weekly working time of employees in Chinese enterprises is 46.4 h, 2.4 h higher than the legal maximum weekly working hours. At present, China is still a developing country, with an aging population, the advantage of labor resources will gradually weaken, and the phenomenon of overtime work may become increasingly severe.

Another noteworthy circumstance in China is the 996 work regime, it derives its name from its requirement that employees work from 9:00 am to 9:00 pm, 6 days per week, i.e. 72 h per week [1]. Some organizations do not enforce the 996 work regime but take a range of measures to encourage employees to work overtime voluntarily, such as reimbursing taxi fares, providing gyms and restaurants to employees who stay late at night [2]. In the long run, the line between mandatory and voluntary overtime has become blurred, and the 996 work regime has become embedded in the so-called corporate culture, where organizations have evolved the overuse of employees into ethical requirements for employees. Long working hours not only harm the physical and mental health of the workforce [3], but also further lead to work-family conflict [4], thereby reducing the effectiveness of the workforce.

This paper holds that the 996 work regime is the result of a long-term interactive game between employers and employees. Whether employees choose to work overtime voluntarily or involuntarily, depending on the work atmosphere and the strategies of employers. In such a dynamic evolution process, employers and employees both cooperate and conflict, and they will choose a strategy conducive to their own development through long-term learning and improvement. In addition, employees are often at a disadvantage in information compared to employers, and the information asymmetry between them will also affect their strategic choices. In this context, we believe evolutionary game theory is an effective method to describe this universal social phenomenon.

The evolutionary game theory originated from the research of evolutionary biology and was largely used to study the competition phenomenon in the process of biological evolution, which is based on the assumption of bounded rationality and limited information [5]. The players are population rather than individuals, and the population keeps learning and imitating in the process of the game, so as to maximize the benefits and reach the equilibrium state in the dynamic process [6]. The evolutionary game theory breaks through the limitation of traditional game theory on the complete rationality of game participants, which is very different from the classical game theory and closer to the actual game situation. So far, evolutionary game theory has been widely used in the field of management, because it has unique advantages in solving the long-term equilibrium problem of bounded rational players' decision-making behavior. It can help solve problems such as a game about the supply and demand of virtual goods between users and developers [7], the choice of coping strategies in social media crisis communication [8], and interactive conflicts between enterprises and government authorities in the regulatory process of sharing economy [9].

As described above, the formation of the 996 work regime can be regarded as a dynamic evolution process between employers and employees, and evolutionary game theory has good applicability here. First of all, we review the causes of voluntary overtime work as well as the relationship between information asymmetry and overtime work.

### Voluntary overtime

Voluntary overtime is defined as working overtime for positive reasons [10]. The concept of involuntary overtime is similar to the loss of control over working hours [11], so the occurrence of involuntary overtime often causes some negative effects, such as high fatigue [12], high levels of depersonalization [13], and low satisfaction [14]. However, overtime is not always explicitly recognized as voluntary or involuntary, and there can be a gray area that is hard to define between voluntary and involuntary overtime [15]. According to existing research, employees may work overtime voluntarily for the following reasons:

Firstly and most crucially, to increase economic income. From the perspective of the economic utility analysis, overtime is a utility maximization decision made by employees based on their ability endowments under a given labor market wage rate, regardless of the group of employees [16]. Under a certain social labor productivity condition, employees are often willing to spend more time on paid work, to increase family income and ensure their career prospects. For example, employees in manual labor and low-skill positions often choose to work overtime voluntarily to increase their economic income [17]. However, in the long run, when the welfare loss caused by the reduction of leisure time is greater than the income increase caused by overtime work, it will bring net welfare loss to employees who work overtime [18].

Secondly, for an organizational culture characterized by overtime. The formation of organizational culture is influenced by the beliefs and values of the leadership [19], which is consolidated through the recruitment and mobility of employees [20] and resocialized through the interaction within the organization [21]. Organizations confuse overtime culture with professional ethics, giving employees the illusion that "no overtime means no effort". In this kind of corporate culture of moral kidnapping, employees' herd mentality will drive them to work overtime voluntarily. Furthermore, organizations often regard whether employees are willing to work overtime as an important criterion to judge their contribution to the organization, leadership potential and promotion [22]. Such a corporate culture blurs the line between voluntary and mandatory overtime, and employees have to put up with the 996 work regime.

Thirdly, for a sense of personal achievement. There are also some employees who are enthusiastic about their work and willing to devote a lot of spare time to their work. According to self-determination theory, when the degree of overtime self-determination is high, employees control overtime behavior with their own will, and the satisfaction of their independent needs brings positive emotions, which alleviates the negative psychological effects of overtime [23]. Furthermore, high self-determination of overtime is often associated with higher work efficiency. Employees can experience a sense of accomplishment and control over their work when productivity increases and work goals are met [24]. Research shows that people with self-driven personalities [25], high levels of conscientiousness, and achievement motivation [26] work overtime more often.

Among them, we believe that salary is the key factor affecting whether employees work overtime. A study has shown that low-paid employees have higher levels of burnout when they work overtime, especially when they work involuntary overtime [27]. However, under the condition of unpaid overtime, employees who work involuntarily overtime are more likely to face job burnout, but higher overtime pay can offset part of the negative effects caused by forced overtime [15]. On the one hand, the longer you work overtime, the more likely you are to feel job burnout. Because commitment to work depletes both physical and mental resources, the longer you work overtime, the more that resource is lost [28]. On the other hand, individuals will feel higher psychological pressure when they are faced with resource loss or resource investment without return [29], and overtime compensation can compensate part of the resources lost in the process of overtime work, which helps reduce the increased psychological pressure caused by overtime work. To conclude, we believe that whether employees choose to work overtime voluntarily and material reward jointly affects their working psychology.

### Information asymmetry

Information asymmetry refers to a relationship in which one party has more or better information than the other [30]. The concept of information asymmetry is widely spread in management research and its existence is the core assumption of organizational frontier theory [31]. In the existing management literature, information asymmetry has been applied to the study of corporate social responsibility performance [32], technical information loss regarding human capital [33], team relationship conflict [34], and enterprise information management ability [35].

To some extent, the information asymmetry between employers and employees also affects overtime behavior. Studies have pointed out that information asymmetry on employee productivity is the cause of low efficiency and long working hours. Since employers cannot observe the true productivity of employees, they use long working hours as a mechanism to screen out productive employees [36]. In addition, when the output of a single employee is difficult to measure, the employer may evaluate the employee's performance in an absolute or relative way and offer potential rewards such as salary increase, bonus, or promotion, which may cause the employee's sense of injustice at work [37]. Secretive pay scales lead more employees to try to boost their pay in the form of overtime[38]. Employees who are treated well and fairly by their employers also tend to prove their commitment by working overtime [39]. However, a study have found that paid overtime and unpaid overtime had no significant effect on salary growth or promotion [40].

### The present study

Existing researches mainly analyze employees' overtime behavior from the perspectives of management, organizational behavior, and psychology [15, 41, 42]. However, few studies have taken information asymmetry into account because it is a difficult variable to quantify in the field of organizational behavior. This paper intends to analyze employees' overtime behavior based on principal-agent theory, comprehensively consider the information asymmetry between employees and employers, and explain the evolutionary learning process of both parties by constructing a dynamic game model of overtime behavior. Through this paper, we want to answer the following questions: (a) In the dynamic game of the employee-employer relationship, what factors affect their respective returns? (b) What are the evolutionary stability strategies of the whole system? and (c) How can both of the parties reach the optimal stable strategy? The conclusion of this study is expected to provide theoretical guidance and practical inspiration for organizations to manage employees' overtime behavior scientifically and rationally improve their psychological state at work.

This paper has made contributions in the following aspects: (a) We use the evolutionary game model to solve problems in organizational behavior, which is a convergence of disciplines. (b) We include information asymmetry into the game model and conduct simulation experiments to quantitatively study the impact of information asymmetry on voluntary overtime work of employees, which is a supplement to existing overtime literature. (c) We use the principal-agent theory to construct the hypothesis and payment matrix, which is an extension of the theory.

## Methods

The most commonly used model in evolutionary game is the two-party game model. In the simplest two-player game, players are given a finite number of strategies, and the game is defined by listing the strategies players use and the benefits they generate [43]. If each player has two strategies to choose from, their strategies can construct a $2\times 2$ symmetric or asymmetric game matrix. According to the relative size of matrix elements, there are four types of games: prisoner’s dilemma, hawk–dove game, stag hunt, and the trivial game with no dilemma [44]. Evolutionary stability strategy (ESS) and replication dynamics equation (RD) are two core concepts of evolutionary game theory. ESS is used to describe a strategy, that is, when the majority of individuals in the population choose a strategy, the group that chooses the mutation strategy cannot invade the group containing the majority of individuals, because it contains fewer individuals, so ESS has good stability in the process of the evolutionary game [45]. RD was proposed by Taylor and Jahnke [46], it refers to the ability of individuals to frequently adjust themselves through imitation, learning, and selection of the current situation [47].

In a two-party game, let ${e}_{1,}{e}_{2,...,}{e}_{m}\in {\Delta }^{m}$ be the $m$ pure strategies of one party, and ${f}_{1,}{f}_{2,...,}{f}_{n}\in {\Delta }^{n}$ be the $n$ pure strategies of the other party. Then, $A,B,C$, and $D$ are the four payoff matrices under the $2\times 2$ strategy matrix. Specifically, $A$ is a $m\times m$ intraspecific payoff matrix, $B$ is a $m\times n$ interspecific payoff matrix, $C$ is a $n\times m$ payoff matrix, and $D$ is a $n\times n$ payoff matrix. The expected returns of both parties of the game depend on their strategies and the strategy pair $(S,T)\in {\Delta }^{m}\times {\Delta }^{n}$ that specify the mean strategies of both parties.

### Definition 1.

[48, 49] $({S}^{*},{T}^{*})\in {\Delta }^{m}\times {\Delta }^{n}$ is an ESS if, for all other $(S,T)$ in ${\Delta }^{m}\times {\Delta }^{n}$,

i. $$(i) S\times (A{S}^{*}+B{T}^{*})\le {S}^{*}\times (A{S}^{*}+B{T}^{*})$$ and $$T\times \left(C{S}^{*}+D{T}^{*}\right)\le {T}^{*}\times \left(C{S}^{*}+D{T}^{*}\right)$$;
ii. if both comparisons in (i) are equalities, then either $S\times (AS+BT)<{S}^{*}\times (AS+BT)$ or $T\times (CS+DT)<{T}^{*}\times (CS+DT)$.

The equilibrium condition (i) maintain that $({S}^{*},{T}^{*})$ is a best strategy pair of ESS. The stability condition (ii) gives at least one party a positive incentive to remain in the ESS component if an alternative best response $\left(S,T\right)$ is considered.

Let ${p}_{k}(t)$ be the frequency at time $t$ of the first party using ${e}_{k}\in {\Delta }^{m}$, and ${q}_{l}(t)$ be the frequency at time $t$ of the other party using ${f}_{l}\in {\Delta }^{n}$, then the replication dynamics equation is [46, 50]

$$\begin{array}{c}{\dot{p}}_{k}={p}_{k}({e}_{k}-p)\times (Ap+Bq)\\ {\dot{q}}_{l}={q}_{l}({f}_{l}-q)\times (Cp+Dq)\end{array}$$

### Problem description

From an organizational point of view, working hour is an important criterion for the game between employees and employers, because the unreasonable working hour and remuneration are important factors for labor conflict. In the labor market, labor supply has exceeded demand for a long time, resulting in serious inequality in labor-capital relations, and employers play a dominant role in the game of labor-capital power.

The principal-agent theory is the application of asymmetric information game theory in economics, it studies the optimal transaction contract under the condition of asymmetric information. Specifically speaking, principal-agent refers to a contractual relationship in which a person or some people (principals) entrust others (agents) to engage in certain activities by the interests of principals, and correspondingly grant agents some decision-making rights [51]. In this contract, the party who takes the initiative to design the contract form is called the principal, while the party who passively accepts the contract form is called the agent. When the principal cannot observe the effort level of the agent, the inertia of the agent will cause the interest loss of the principal. In this case, the principal will design the corresponding incentive mechanism to induce the agent to improve the effort level [52].

According to principal-agent theory, the employer can be regarded as the principal and the employee as the agent in our game. Overtime work behavior is the result of a long-term dynamic game between employees and employers. The unequal status of labor and capital causes information asymmetry between employees and employers, and employers play a dominant role. In order to maximize corporate profits, employers have a strong incentive to manipulate employees' working hours and turn it into reality. However, due to information asymmetry, it is difficult for employers to determine whether employees are working overtime effectively, and thus pay employees at all levels equally for overtime. Similarly, employees who choose to volunteer overtime also have a hard time judging the authenticity of positive messages from employers, which tend to avoid negative messages in order to increase employee motivation and loyalty. In principal-agent issues, information asymmetry after a contract is signed can lead to moral hazard [53], so in our game, both employers and employees face the problem of moral hazard. In addition, research has shown that trust and reciprocity between players are more important than incentives in principal-agent problems [54], thus we believe that mutual trust between employees and employers will produce synergistic benefits.

### Model assumptions

As have discussed above, we assume that both employers and employees are bounded rational economic individuals. When they are faced with incomplete information, their strategies are not optimal at the beginning. But as time goes by, they gradually find the optimal strategy for themselves through continuous learning and trial. The purpose of employees is to maximize their labor utility, while the purpose of employers is to maximize profits.

During the dynamic game between employees and employers, both parties have two choice strategies. Employees can be divided into working overtime voluntarily or involuntarily. Assume that the ratio of employees who choose voluntary overtime strategy is $x$, and the ratio of employees who choose involuntary overtime strategy is $1-x$. Employers can choose to give overtime pay and not give overtime pay. Assume that the ratio of employers who choose to pay overtime is $y$, and the ratio of employers who choose not to pay overtime is $1-y$. Apparently, both parties will consume certain resources, such as time, energy and material, etc. It is assumed that the resources invested by employees and employers are ${R}_{i}$ and ${R}_{j}$ respectively. One of the game strategies is (Involuntarily, Not pay), in this case, the benefits of employees and employers are ${\Pi }_{i}$ and ${\Pi }_{j}$, respectively. The optimal strategy is (Voluntarily, Pay), both parties of the game trust each other and generate additional synergistic benefits, such as material rewards, good reputation and social recognition. Assume that the employee's trust coefficient to the employer is ${\theta }_{i}$, and the employer's trust coefficient to the employee is ${\theta }_{j}$. Then the synergistic benefits earned by employees and employers are ${\theta }_{i}{R}_{i}$ and ${\theta }_{j}{R}_{j}$, respectively. Furthermore, suppose that the information asymmetry coefficient of employee is ${\alpha }_{i}$, that of employers is ${\alpha }_{j}$, and ${\alpha }_{i}>{\alpha }_{j}>0$. Information asymmetry between employees and employers will result in a certain cost, the cost paid by employees is ${\alpha }_{i}{R}_{i}$, the cost paid by employers is ${\alpha }_{j}{R}_{j}$. Finally, assume that the moral hazard coefficient of employees is ${\beta }_{i}$ and that of employers is ${\beta }_{j}$. If an employee chooses the involuntary overtime strategy and the employer offers overtime payment, the employee can obtain additional moral hazard benefit ${\beta }_{i}{R}_{j}$ from the employer. On the other hand, if the employer chooses the strategy of not offering overtime payment and the employee chooses to work overtime voluntarily, then the employer can obtain additional moral hazard benefit ${\beta }_{j}{R}_{i}$ from the employee.

Table 1 shows all the parameters used in our game and their descriptions.

**Table 1:** Description of parameters

Parameters	Descriptions
$x$	The ratio of employees who choose voluntary overtime strategy
$y$	The ratio of employers who choose to pay overtime
${\Pi }_{i}$	The initial benefits of employees
${\Pi }_{j}$	The initial benefits of employers
${R}_{i}$	The resources invested by employees
${R}_{j}$	The resources invested by employers
${\theta }_{i}$	The employee's trust coefficient to the employer
${\theta }_{j}$	The employer's trust coefficient to the employee
${\alpha }_{i}$	The information asymmetry coefficient of employees
${\alpha }_{j}$	The information asymmetry coefficient of employers
${\beta }_{i}$	The moral hazard coefficient of employees
${\beta }_{j}$	The moral hazard coefficient of employers

### Payment matrix

Based on the above assumptions, we can construct a payment matrix about the employee-employer relationship of overtime behavior, as shown in Table 2.

**Table 2:** Payment matrix between employees and employers

		Employee $(i)$	
		Voluntarily	Involuntarily
Employer $(j)$	Pay $(y)$	$$(\begin{array}{c}{\Pi }_{i}-{\alpha }_{i}{R}_{i}+{\theta }_{i}{R}_{i},\\ {\Pi }_{j}-{\alpha }_{j}{R}_{j}+{\theta }_{j}{R}_{j}\end{array})$$	$({\Pi }_{i}+{\beta }_{i}{R}_{j},{\Pi }_{j}-{\alpha }_{j}{R}_{j})$
	Not pay $(1-y)$	$({\Pi }_{i}-{\alpha }_{i}{R}_{i},{\Pi }_{j}+{\beta }_{j}{R}_{i})$	$({\Pi }_{i},{\Pi }_{j})$

**Case 1** (Involuntarily, Not pay). Employees have to work overtime involuntarily without pay, in which case the benefits of employees and employers are ${\Pi }_{i}$ and ${\Pi }_{j}$ respectively.

**Case 2** (Voluntarily, Not pay). Employees who volunteer to work overtime pay extra costs ${\alpha }_{i}{R}_{i}$ due to information asymmetry. However, employers can benefit ${\beta }_{j}{R}_{i}$ from employees who volunteer to work overtime because they get free labor at no extra cost.

**Case 3** (Involuntarily, Pay). Employers cannot judge the overtime efficiency of the employees who work overtime involuntarily, but they offer the same remuneration to employees at the same level, so the employees can get extra benefits ${\beta }_{i}{R}_{j}$ from employers, while employers have to pay the cost ${\alpha }_{j}{R}_{j}$ caused by information asymmetry.

**Case 4** (Voluntarily, Pay). Due to information asymmetry, both parties have to pay a certain cost of ${\alpha }_{i}{R}_{i}$ and ${\alpha }_{j}{R}_{j}$. In addition, it is a harmonious state in which both parties trust each other and they can gain additional benefits $\theta R$ due to trust.

## Results

### Evolutionary stability analysis

According to Table 2, at a $t$ moment, the expected returns of an employee’s choice of voluntary overtime are:

1 $$\begin{array}{c}E\left({A}_{1}\right)=y\left({\Pi }_{i}-{\alpha }_{i}{R}_{i}+{\theta }_{i}{R}_{i}\right)+\left(1-y\right)\left({\Pi }_{i}-{\alpha }_{i}{R}_{i}\right)\end{array}$$

The expected returns of an employee’s choice of involuntary overtime are:

2 $$\begin{array}{c}E\left({A}_{2}\right)=y\left({\Pi }_{i}+{\beta }_{i}{R}_{j}\right)+\left(1-y\right){\Pi }_{i}\end{array}$$

The employee’s average expected returns can be denoted by:

3 $$\begin{aligned}\overline{E }\left(A\right) =&\, xE\left({A}_{1}\right)+\left(1-x\right)E\left({A}_{2}\right)\\ = \,& x\left[y\left({\Pi }_{i}-{\alpha }_{i}{R}_{i}+{\theta }_{i}{R}_{i}\right)+\left(1-y\right)\left({\Pi }_{i}-{\alpha }_{i}{R}_{i}\right)\right]\\ &+\left(1-x\right)\left[y\left({\Pi }_{i}+{\beta }_{i}{R}_{j}\right)+\left(1-y\right){\Pi }_{i}\right]\end{aligned}$$

The replicated dynamic equation of the employee can be expressed as follows:

4 $$\begin{aligned}f\left(x\right) & = \frac{dx}{dt} = x\left[E\left({A}_{1}\right)-\overline{E }\left(A\right)\right]\\ &= x\left(1-x\right)\left[E\left({A}_{1}\right)-E\left({A}_{2}\right)\right]\\ &=x\left(1-x\right)\left[y\left({\theta }_{i}{R}_{i}-{\beta }_{i}{R}_{j}\right)-{\alpha }_{i}{R}_{i}\right]\end{aligned}$$

Similarly, the expected returns of an employer’s choice of pay strategy and not pay strategy are as follows:

5 $$\begin{array}{c}E\left({B}_{1}\right)=x\left({\Pi }_{j}-{\alpha }_{j}{R}_{j}+{\theta }_{j}{R}_{j}\right)+\left(1-x\right)\left({\Pi }_{j}-{\alpha }_{j}{R}_{j}\right)\end{array}$$

6 $$\begin{array}{c}E\left({B}_{2}\right)=x\left({\Pi }_{j}+{\beta }_{j}{R}_{i}\right)+\left(1-x\right){\Pi }_{j}\end{array}$$

Accordingly, the employer’s average expected returns can be denoted by:

7 $$\begin{aligned}\overline{E }\left(B\right)&= yE\left({B}_{1}\right)+\left(1-y\right)E\left({B}_{2}\right)\\ &=y\left[x\left({\Pi }_{j}-{\alpha }_{j}{R}_{j}+{\theta }_{j}{R}_{j}\right)+\left(1-x\right)\left({\Pi }_{j}-{\alpha }_{j}{R}_{j}\right)\right]\\ &\quad +\left(1-y\right)\left[x\left({\Pi }_{j}+{\beta }_{j}{R}_{i}\right)+\left(1-x\right){\Pi }_{j}\right]\end{aligned}$$

Further, the replicated dynamic equation of the employer can be expressed as follows:

8 $$\begin{array}{c}\begin{array}{c}f\left(y\right)=\frac{dy}{dt}=y\left[E\left({B}_{1}\right)-\overline{E }\left(B\right)\right]\\ =y\left(1-y\right)\left[E\left({B}_{1}\right)-E\left({B}_{2}\right)\right]\\ \quad \quad =y\left(1-y\right)\left[x\left({\theta }_{j}{R}_{j}-{\beta }_{j}{R}_{i}\right)-{\alpha }_{j}{R}_{j}\right]\end{array}\end{array}$$

Equilibrium points $(x,y)$ are when both dynamic equations are equal to zero. According to Eqs. (4) and (8), we can obtain the following two theorems:

#### Theorem 1.

*Five equilibrium points can be acquired, namely* ${E}_{1}(\mathrm{0,0})$, ${E}_{2}(\mathrm{1,0})$, ${E}_{3}(\mathrm{0,1})$, ${E}_{4}(\mathrm{1,1})$, *and* ${E}_{5}({x}^{*},{y}^{*})$, *where:*

$${x}^{*}=\frac{{\alpha }_{j}{R}_{j}}{{\theta }_{j}{R}_{j}-{\beta }_{j}{R}_{i}},{ y}^{*}=\frac{{\alpha }_{i}{R}_{i}}{{\theta }_{i}{R}_{i}-{\beta }_{i}{R}_{j}}$$

#### Proof.

Substituting the five equilibrium points into Eqs. (4) and (8), it is easy to observe that all of them satisfy $f(x)=0$ and $f(y)=0$.

Theorem 1 reveals all equilibrium points according to the replication dynamic equations Eqs. (4) and (8), but whether these equilibrium points satisfy ESS needs further analysis. According to the definition of ESS in Definition 1, we can derive Theorem 2:

#### Theorem 2

*The equilibrium point* ${E}_{1}(\mathrm{0,0})$ *is the ESS, the equilibrium point* ${E}_{5}({x}^{*},{y}^{*})$ *is a saddle point, while the other three equilibrium points need to be discussed on a case-by-case basis*.

#### Proof

According to Friedman [5], the stability of equilibrium points can be obtained by analyzing the local stability of the Jacobian matrix, which can be defined by:

9 $$\begin{array}{c}J=\left[\begin{array}{cc}\frac{df\left(x\right)}{dx}& \frac{df\left(x\right)}{dy}\\ \frac{df\left(y\right)}{dx}& \frac{df\left(y\right)}{dy}\end{array}\right]=\left[\begin{array}{cc}{a}_{11}& {a}_{12}\\ {a}_{21}& {a}_{22}\end{array}\right]\end{array}$$

where:

$$\begin{array}{l}{a}_{11}=(1-2x)[y\left({\theta }_{i}{R}_{i}-{\beta }_{i}{R}_{j}\right)-{\alpha }_{i}{R}_{i}]\\ {a}_{12}=x(1-x)({\theta }_{i}{R}_{i}-{\beta }_{i}{R}_{j})\\ {a}_{21}=y(1-y)({\theta }_{j}{R}_{j}-{\beta }_{j}{R}_{i})\\ { a}_{22}=(1-2y)[x\left({\theta }_{j}{R}_{j}-{\beta }_{j}{R}_{i}\right)-{\alpha }_{j}{R}_{j}]\end{array}$$

The expression of the determinant and trace of the Jacobian matrix at five equilibrium points is shown in Table 3.

**Table 3:** The expression of the determinant and trace at five equilibrium points

Equilibrium points	det(${\varvec{J}}$)	tr(${\varvec{J}}$)
${E}_{1}(\mathrm{0,0})$	$({\alpha }_{i}{R}_{i})\times ({\alpha }_{j}{R}_{j})$	$-({\alpha }_{i}{R}_{i}+{\alpha }_{j}{R}_{j})$
${E}_{2}(\mathrm{1,0})$	${\alpha }_{i}{R}_{i}\times ({\theta }_{j}{R}_{j}-{\beta }_{j}{R}_{i}-{\alpha }_{j}{R}_{j})$	${\alpha }_{i}{R}_{i}+({\theta }_{j}{R}_{j}-{\beta }_{j}{R}_{i}-{\alpha }_{j}{R}_{j})$
${E}_{3}(\mathrm{0,1})$	${\alpha }_{j}{R}_{j}\times ({\theta }_{i}{R}_{i}-{\beta }_{i}{R}_{j}-{\alpha }_{i}{R}_{i})$	${\alpha }_{j}{R}_{j}+({\theta }_{i}{R}_{i}-{\beta }_{i}{R}_{j}-{\alpha }_{i}{R}_{i})$
${E}_{4}(\mathrm{1,1})$	$\left({\theta }_{i}{R}_{i}-{\beta }_{i}{R}_{j}-{\alpha }_{i}{R}_{i}\right)\times$ $({\theta }_{j}{R}_{j}-{\beta }_{j}{R}_{i}-{\alpha }_{j}{R}_{j})$	$-[({\theta }_{i}{R}_{i}-{\beta }_{i}{R}_{j}-{\alpha }_{i}{R}_{i})+$ $({\theta }_{j}{R}_{j}-{\beta }_{j}{R}_{i}-{\alpha }_{j}{R}_{j})]$
${E}_{5}({x}^{*},{y}^{*})$	$-\left({\alpha }_{i}{R}_{i}\right)\times \left({\alpha }_{j}{R}_{j}\right)$ $\times (1-{x}^{*})\times (1-{y}^{*})$	0

It is obvious that $({\alpha }_{i}{R}_{i})\times ({\alpha }_{j}{R}_{j})$>0 and $-\left({\alpha }_{i}{R}_{i}+{\alpha }_{j}{R}_{j}\right)<0$ are all true in any cases, thus ${E}_{1}(\mathrm{0,0})$ is the ESS. Likewise, $-\left({\alpha }_{i}{R}_{i}\right)\times \left({\alpha }_{j}{R}_{j}\right)\times \left(1-{x}^{*}\right)\times \left(1-{y}^{*}\right)<0$ is true under any circumstances, thus ${E}_{5}({x}^{*},{y}^{*})$ is a saddle point. However, the determinant and trace size of the other three equilibrium points need to be determined by discussing the sign fraction of the parameters.

Theorem 2 indicates that there must be an ESS in the system for two parties to establish a stable relationship. Both parties of the game can constantly adjust from the initial state so as to achieve the optimal revenue, and the evolution path is dependent.

In order to further determine the evolutionary stability strategy of the system, we substitute the above five equilibrium points into the Jacobian matrix to calculate its determinant and trace for local stability analysis under different constraints. See Table 4 for details. The equilibrium strategy under different constraints is only an ideal state, but it has important implications for organizational management. We put forward the following propositions:

**Table 4:** Local stability analysis of different scenarios at each equilibrium point

Scenarios	Constraints	Points	det(${\varvec{J}}$)	tr(${\varvec{J}}$)	Results
Scenario 1	$$\left\{\begin{array}{c}{\theta }_{i}{R}_{i}-{\beta }_{i}{R}_{j}<{\alpha }_{i}{R}_{i}\\ {\theta }_{j}{R}_{j}-{\beta }_{j}{R}_{i}<{\alpha }_{j}{R}_{j}\end{array}\right.$$	${E}_{1}$	$+$	$-$	ESS
		${E}_{2}$	$-$	Uncertain	Saddle point
		${E}_{3}$	$-$	Uncertain	Saddle point
		${E}_{4}$	$+$	$+$	Unstable
		${E}_{5}$	$-$	0	Saddle point
Scenario 2	$$\left\{\begin{array}{c}{\theta }_{i}{R}_{i}-{\beta }_{i}{R}_{j}>{\alpha }_{i}{R}_{i}\\ {\theta }_{j}{R}_{j}-{\beta }_{j}{R}_{i}>{\alpha }_{j}{R}_{j}\end{array}\right.$$	${E}_{1}$	$+$	$-$	ESS
		${E}_{2}$	$+$	$+$	Unstable
		${E}_{3}$	$+$	$+$	Unstable
		${E}_{4}$	$+$	$-$	ESS
		${E}_{5}$	$-$	0	Saddle point

#### Proposition 1

*When the benefits gained through moral hazard is greater than the difference between the synergy benefit generated by cooperative trust and the cost of information asymmetry, the game will stabilize at (Involuntarily, Not pay).*

#### Proof

**Fig. 1:**
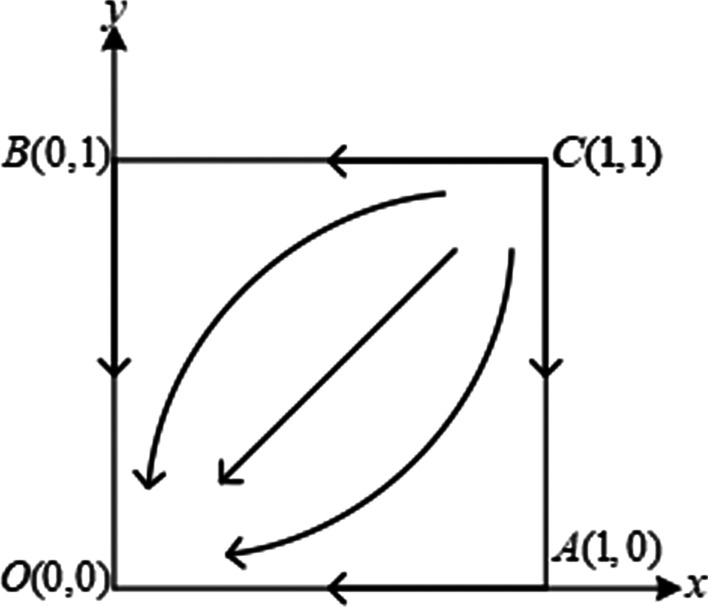
Evolution phase diagram of the system under scenario 1

When ${\theta }_{i}{R}_{i}-{\beta }_{i}{R}_{j}<{\alpha }_{i}{R}_{i}$ and${\theta }_{j}{R}_{j}-{\beta }_{j}{R}_{i}<{\alpha }_{j}{R}_{j}$, the system has a unique evolutionary stable point ${E}_{1}(\mathrm{0,0})$, three saddle points, and one unstable point. This means that no matter what the initial conditions of the system are, it will evolve from the unstable point through the saddle point to the only stable state. The phase changes of system evolution are shown in Fig. 1.

#### Proposition 2

*When the benefit gained through moral hazard is less than the difference between the synergy benefit generated by cooperative trust and the cost of information asymmetry, there are two sets of equilibrium strategies: (Voluntarily, Pay) and (Involuntarily, Not pay).*

#### Proof.

**Fig. 2:**
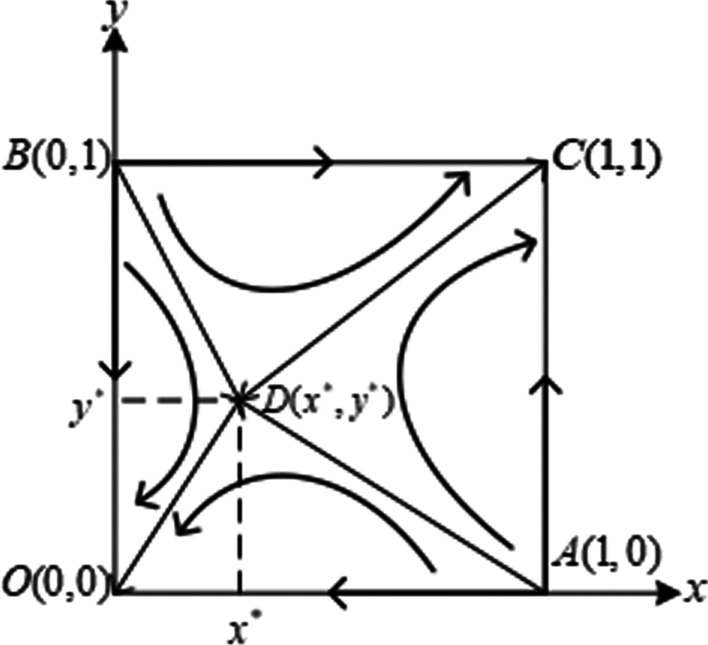
Evolution phase diagram of the system under scenario 2

When ${\theta }_{i}{R}_{i}-{\beta }_{i}{R}_{j}>{\alpha }_{i}{R}_{i}$ and ${\theta }_{j}{R}_{j}-{\beta }_{j}{R}_{i}>{\alpha }_{j}{R}_{j}$, the system has two evolutionary stable points ${E}_{1}(\mathrm{0,0})$ and ${E}_{4}(\mathrm{1,1})$, two unstable points ${E}_{2}(\mathrm{1,0})$ ${E}_{3}(\mathrm{0,1})$, and one saddle point ${E}_{5}({x}^{*},{y}^{*})$. As can be seen from Fig. 2, the broken lines $BD$ and $DA$ connected by unstable points and saddle points constitute the dividing line of system convergence to different equilibrium points. The $ADBC$ part on the upper right of the broken line will converge to point equilibrium $(\mathrm{1,1})$ of the entire evolutionary system under the action of evolutionary stability strategy $DC$, while the $ADBO$ part at the lower left of the broken line converges to the equilibrium point $(\mathrm{0,0})$ of the entire evolutionary system under the action of evolutionary stability strategy $DO$. These two different evolutionary stability strategies coexist, but their properties are quite different.

#### Proposition 3.

*Specifically, when the difference between the synergy benefit generated by trust and the cost of information asymmetry is greater than twice the moral hazard benefit, the game will stabilize at (Voluntarily, Pay).*

#### Proof.

When ${\theta }_{i}{R}_{i}-{\beta }_{i}{R}_{j}>{2\alpha }_{i}{R}_{i}$ and ${\theta }_{j}{R}_{j}-{\beta }_{j}{R}_{i}>{2\alpha }_{j}{R}_{j}$, which refers to ${\alpha }_{i}{R}_{i}/({\theta }_{i}{R}_{i}-{\beta }_{i}{R}_{j})<1/2$ and ${\alpha }_{j}{R}_{j}/({\theta }_{j}{R}_{j}-{\beta }_{j}{R}_{i})<1/2$, thus ${x}^{*}<1/2$ and ${y}^{*}<1/2$. The area of $ADBC$ in Fig. 2 will be greater than the area of $ADBO$, the final strategy of the system will stabilize at $C(\mathrm{1,1})$.

### Simulation experiments

Through the theoretical analysis demonstrated above, two evolutionary game stability strategies have been recognized, which can be acquired when corresponding constraints are satisfied. In order to intuitively reveal the evolutionary trajectories of employees and employers, along with their sensitivity to each parameter, this section intends to simulate the model based on the three constraints and replication dynamics equations by using MATLAB.

### The number of resources consumed

**Scenario 1.** We set ${\theta }_{i}={\theta }_{j}=0.8$, ${\beta }_{i}={\beta }_{j}=0.6$, ${\alpha }_{i}=0.4$, and ${\alpha }_{j}=0.3$, then we can have

$$\left\{\begin{array}{c}0.8{R}_{i}-0.6{R}_{j}<0.4{R}_{i}\\ 0.8{R}_{j}-0.6{R}_{i}<0.3{R}_{j}\end{array}\right.\Rightarrow \left\{\begin{array}{c}0.4{R}_{i}<0.6{R}_{j}\\ 0.5{R}_{j}<0.6{R}_{i}\end{array}\right.\Rightarrow \frac{5}{6}{R}_{j}<{R}_{i}<\frac{3}{2}{R}_{j}$$

Here we simulate values ${R}_{j}=\mathrm{6,12,18,24}$ respectively to observe their impact on the system.

**Fig. 3:**
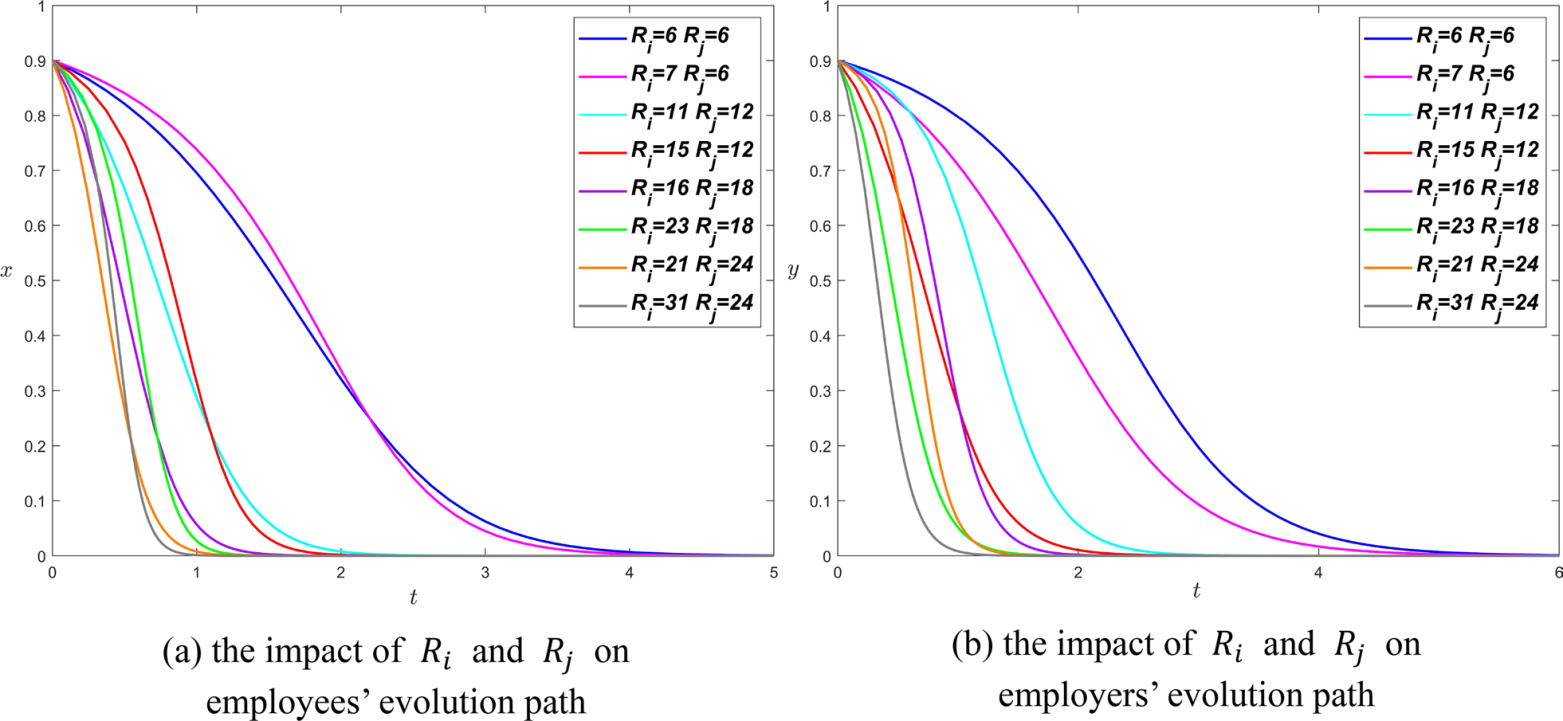
The influence curve of resource consumed on evolution results under scenario 1

In Fig. 3, as ${R}_{i}$ and ${R}_{j}$ increases, the curves are closer to $y$-axis, which means the increase of the two parameters will help shorten the time for the system to reach stability. Moreover, the influence of ${R}_{j}$ is significantly greater than that of ${R}_{i}$ for both parties. Thus, the Proposition 1 is confirmed from Fig. 3. In addition, the increase of resources invested by both parties of the game will help the system stabilize at (Involuntarily, Not pay), especially the increase of employers’ resources.

**Scenario 2.** We set ${\theta }_{i}={\theta }_{j}=0.9$, ${\beta }_{i}={\beta }_{j}=0.2$, ${\alpha }_{i}=0.5$, and ${\alpha }_{j}=0.2$, therefore

$$\left\{\begin{array}{c}0.9{R}_{i}-0.2{R}_{j}>0.5{R}_{i}\\ 0.9{R}_{j}-0.2{R}_{i}>0.2{R}_{j}\end{array}\right.\Rightarrow \left\{\begin{array}{c}0.4{R}_{i}>0.2{R}_{j}\\ 0.7{R}_{j}>0.2{R}_{i}\end{array}\right.\Rightarrow \frac{1}{2}{R}_{j}<{R}_{i}<\frac{7}{2}{R}_{j}$$

**Fig. 4:**
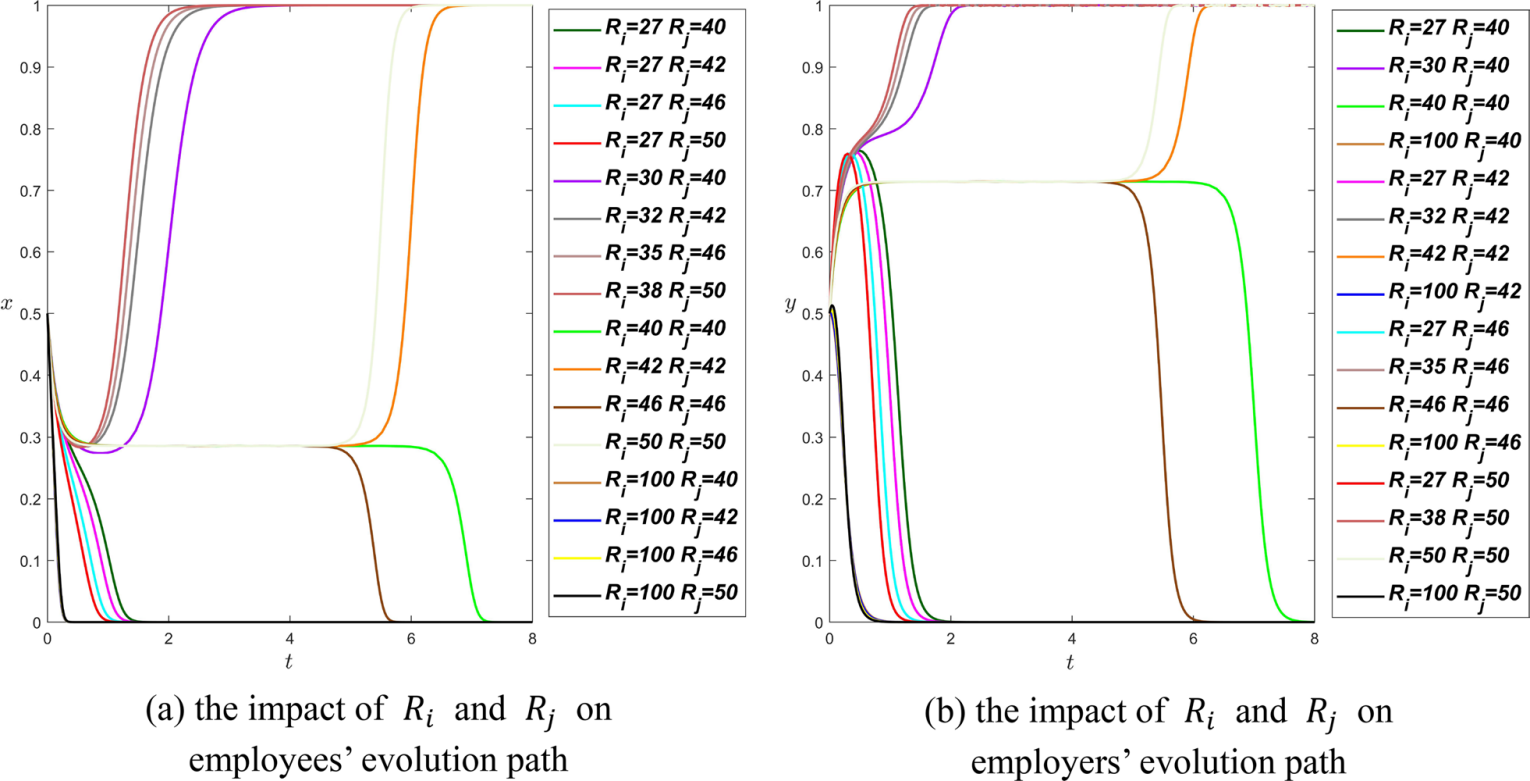
The influence curve of resource consumed on evolution results under scenario 2

Then we assign values ${R}_{j}=\mathrm{40,42,46,50}$ respectively to in Fig. 4.

Obviously, the Proposition 2 is confirmed according to the trend of the curves in Fig. 4. The effect of ${R}_{i}$ and ${R}_{j}$ on the evolutionary path of both parties is relatively complex in scenario 2. There are roughly three situations: (1) if ${R}_{i}={R}_{j}$, both parties of the game will undergo a long linear path evolution and then stabilize. (2) if ${R}_{i}$ is much smaller than ${R}_{j}$ or ${R}_{i}$ is much larger than ${R}_{j}$, both parties of the game will reach equilibrium in a short time at $(\mathrm{0,0})$. (3) When ${R}_{i}$ is close to but not equal to ${R}_{j}$, both parties will finally reach equilibrium at $(\mathrm{1,1})$ in a short time. In addition, these situations are also affected by the values of the other parameters we set. In order to make employees and employers reach equilibrium faster in the long-term dynamic game evolution process, the number of resources invested by employees and employers should be similar or even the same.

### Information asymmetry coefficient

**Scenario 1.** We set ${R}_{i}={R}_{j}=4$, ${\theta }_{i}={\theta }_{j}=0.9$, ${\beta }_{i}={\beta }_{j}=0.8$, then we can have

$$\left\{\begin{array}{l}0.9\times 4-0.8\times 4<{\alpha }_{i}\times 4\\ 0.9\times 4-0.8\times 4<{\alpha }_{j}\times 4\\ {\alpha }_{i}<{\alpha }_{j}\end{array}\right.\Rightarrow {\alpha }_{i}>{\alpha }_{j}>0.1$$

Therefore, we set ${\alpha }_{i}=\mathrm{0.3,0.4,0.5,0.6,0.7,0.8,0.9}$, ${\alpha }_{j}=\mathrm{0.2,0.3,0}.\mathrm{4,0.5,0.6,0.7,0.8}$ to simulate the system.

When the value of ${\alpha }_{i}$ increases from 0.3 to 0.7, the evolutionary path curve of employees is closer to the $y$-axis. That is to say, the increase in ${\alpha }_{i}$ will help short the time it took employees to reach equilibrium. When ${\alpha }_{i}$ value is equal to 0.8 and 0.9, the evolution curves no longer follow the above rules and deviate far from the $y$-axis, which indicates that when the information asymmetry coefficient increases to a certain extent, the system will take a long time to stabilize.

Under the same conditions of other parameters, the curve in figure (a) can approach the $x$-axis at $t=15$, but most of the curves in figure (b) approach the $x$-axis until $t=90$, or even $t=150$. It is suggested that employers take much longer than employees to reach equilibrium when other parameters being equal. In figure (b), the two curves with the longest evolution time are ${\alpha }_{i}=0.8,{\alpha }_{j}=0.2$ and ${\alpha }_{i}=0.9,{\alpha }_{j}=0.2$. The difference in information asymmetry coefficient between the two groups of data is relatively large, and the information asymmetry coefficient of employees is much larger than that of employers. However, if ${\alpha }_{j}$ value is fixed at 0.2, ${\alpha }_{i}=\mathrm{0.3,0.4,0.5,0.6,0.7}$, the evolution curves are very close to the $y$-axis, and equilibrium can be reached at $t=10$. There are also two more irregular curves, ${\alpha }_{i}=0.8,{\alpha }_{j}=0.7$ and ${\alpha }_{i}=0.9,{\alpha }_{j}=0.8$, which represent large asymmetry coefficients on both parties, and employers can easily reach the equilibrium point in both cases.

**Fig. 5:**
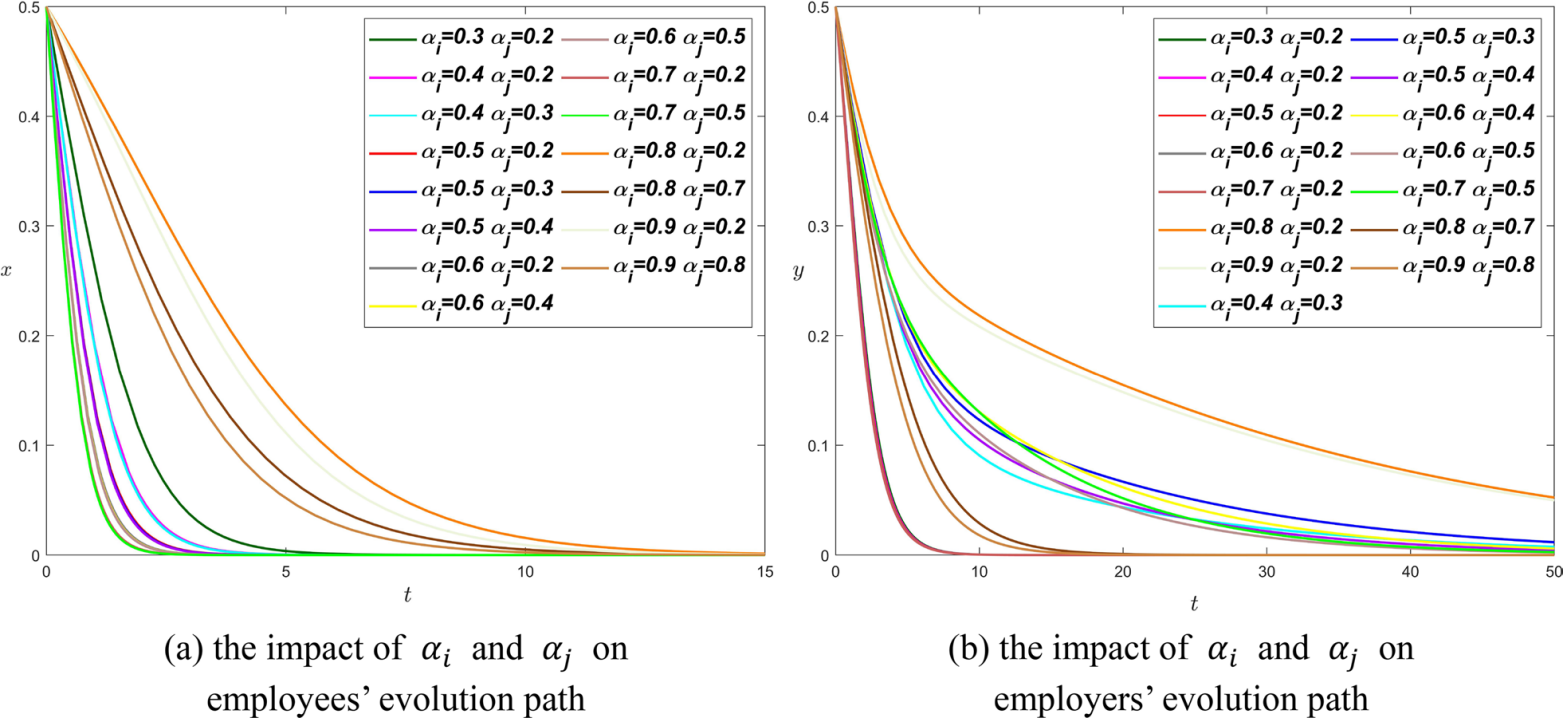
The influence curve of information asymmetry coefficient on evolution results under scenario 1

As a whole, the information asymmetry coefficient has a great influence on the evolution time of both parties, and the Proposition 1 can also be confirmed from Fig. 5. In order to stabilize the system, the difference in information asymmetry coefficient between employees and employers should not be too large. ${\alpha }_{i}$ and ${\alpha }_{j}$ are either small or large, which indicates that employees and employers should have similar information about overtime strategies. However, the information asymmetry coefficient of employees is often greater than that of employers in real life. Hence, employers have the obligation to share information with employees in a timely manner to narrow the information asymmetry gap, so as to achieve a win–win situation.

**Scenario 2.** We set ${R}_{i}={R}_{j}=4$, ${\theta }_{i}={\theta }_{j}=0.8$, ${\beta }_{i}={\beta }_{j}=0.2$, it follows that

$$\left\{\begin{array}{l}0.8\times 4-0.2\times 4>{\alpha }_{i}\times 4\\ 0.8\times 4-0.2\times 4>{\alpha }_{j}\times 4\\ {\alpha }_{i}<{\alpha }_{j}\end{array}\right.\Rightarrow 0.6>{\alpha }_{i}>{\alpha }_{j}$$

**Fig. 6:**
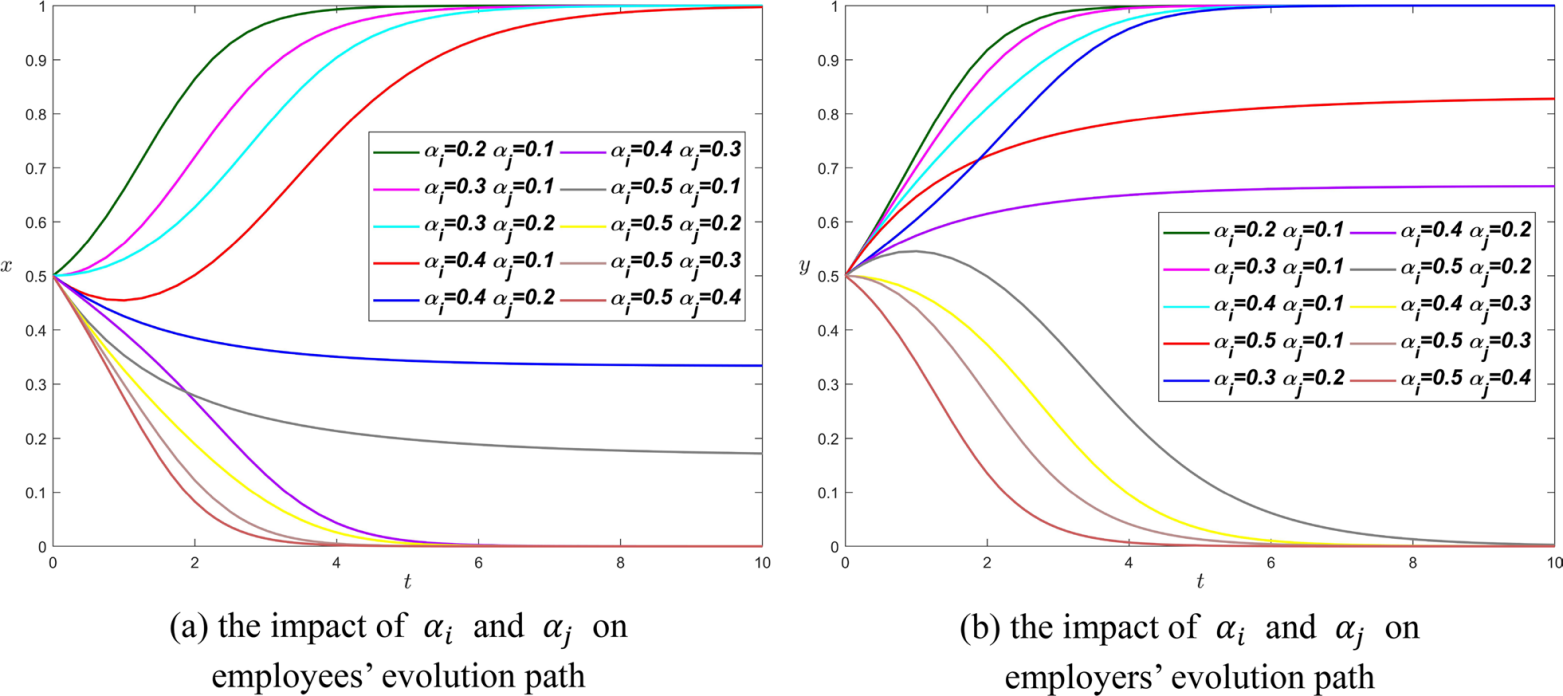
The influence curve of information asymmetry coefficient on evolution results under scenario 2

In Fig. 6, we change the value of ${\alpha }_{i}$ from 0.2 to 0.5 and the value of ${\alpha }_{j}$ from 0.1 to 0.4. The Proposition 2 is confirmed according to Fig. 6.

On the left, when ${\alpha }_{i}$ is fixed, the smaller ${\alpha }_{j}$ is, the faster $x\to 1$; When ${\alpha }_{j}$ is fixed, the smaller ${\alpha }_{i}$ is, the faster $x\to 1$. In the lower part of figure (a), when ${\alpha }_{i}$ is fixed, the larger ${\alpha }_{j}$ is, the faster $x\to 0$; When ${\alpha }_{j}$ is fixed, the bigger ${\alpha }_{i}$ is, the faster $x\to 0$. In addition, there are two abnormal curves ${\alpha }_{i}=0.4,{\alpha }_{j}=0.2$ and ${\alpha }_{i}=0.5,{\alpha }_{j}=0.1$, which approach $x=1$ at around $t=75$ and $t=125$ respectively.

The right picture and the left picture have similar patterns. For the top four curves, the smaller the ${\alpha }_{j}$ is when the ${\alpha }_{i}$ is fixed, or the smaller the ${\alpha }_{i}$ is when the ${\alpha }_{j}$ is fixed, the easier $y\to 1$. The bottom four curves take longer to reach the equilibrium point than the top four, and the larger the ${\alpha }_{j}$ when the ${\alpha }_{i}$ is fixed or the larger the ${\alpha }_{i}$ when the ${\alpha }_{j}$ is fixed, the easier $y\to 0$. Similarly, there are two anomalous curves ${\alpha }_{i}=0.4,{\alpha }_{j}=0.2$ and ${\alpha }_{i}=0.5,{\alpha }_{j}=0.1$ that approach $x=1$ at around $t=75$ and $t=115$, respectively.

In particular, when ${\alpha }_{i}=0.2,{\alpha }_{j}=0.1$, which satisfies ${\theta }_{i}{R}_{i}-{\beta }_{i}{R}_{j}>{2\alpha }_{i}{R}_{i}$ and ${\theta }_{j}{R}_{j}-{\beta }_{j}{R}_{i}>{2\alpha }_{j}{R}_{j}$, the system stabilizes at (Voluntarily, Pay) from Fig. 6, thus, Proposition 3 is confirmed.

By summarizing the curve rules of the two graphs, we can find that the information asymmetry coefficients of the curves in the upper part of the graph are relatively small, and both parties of the game can reach the equilibrium point faster with the decrease of the two parameters. The information asymmetry coefficients of curves in the lower part of the graph are slightly larger, and both parties of the game reach the equilibrium point faster with the increase of the two parameters. The practical significance of scenario 2 is similar to scenario 1, that is, the information asymmetry coefficient gap between employees and employers should not be too large, and only when ${\alpha }_{i}$ and ${\alpha }_{j}$ are both small or large, can both parties achieve equilibrium. However, in scenario 2, we expect both parties to choose the equilibrium point $(\mathrm{1,1})$, therefore, the information asymmetry coefficients of both parties should be small and the gap between them is not too large.

### Trust coefficient

**Scenario 1.** We set ${R}_{i}={R}_{j}=4$, ${\beta }_{i}={\beta }_{j}=0.3$, ${\alpha }_{i}=0.4$, and ${\alpha }_{j}=0.3$, then

$$\left\{\begin{array}{c}0<4\times {\theta }_{i}-0.3\times 4<0.4\times 4\\ 0<4\times {\theta }_{j}-0.3\times 4<0.3\times 4\end{array}\right.\Rightarrow \left\{\begin{array}{c}0.3<{\theta }_{i}<0.7\\ 0.3<{\theta }_{j}<0.6\end{array}\right.$$

**Fig. 7:**
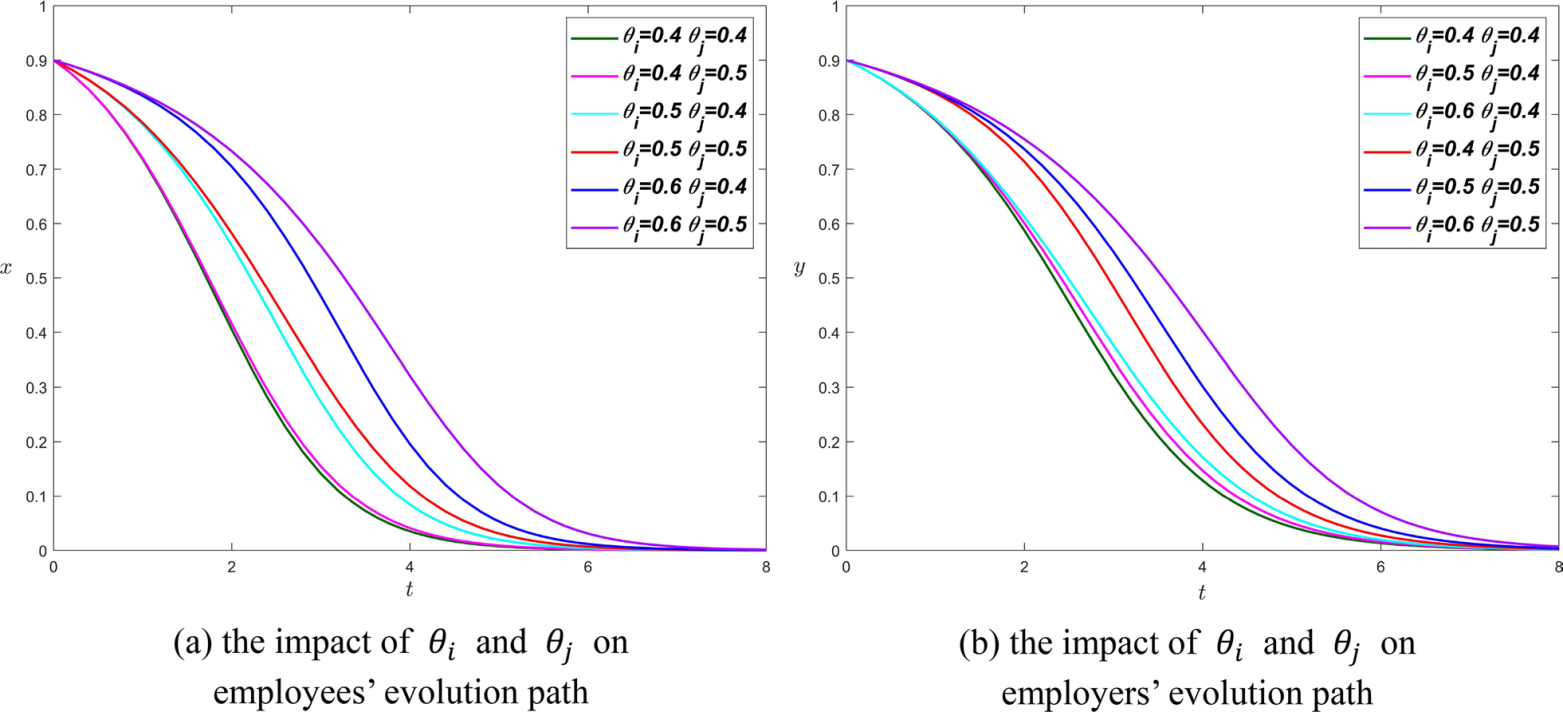
The influence curve of trust coefficient on evolution results under scenario 1

In scenario 1, the impact of the trust coefficient on the evolution path of employees and employers is basically the same. The evolution curves of both parties all reach the equilibrium point until t = 8. The increase of ${\theta }_{i}$ and ${\theta }_{j}$ makes the evolution curves of employees and employers deviate from the $y$-axis, which also prolongs the time for the system to reach the equilibrium point. The Proposition 1 is also confirmed from Fig. 7.

**Scenario 2.** We set ${R}_{i}={R}_{j}=4$, ${\beta }_{i}={\beta }_{j}=0.3$, ${\alpha }_{i}=0.3$, and ${\alpha }_{j}=0.2$, then we can have

$$\left\{\begin{array}{c}4\times {\theta }_{i}-0.3\times 4>0.3\times 4\\ 4\times {\theta }_{j}-0.3\times 4>0.2\times 4\end{array}\right.\Rightarrow \left\{\begin{array}{c}{\theta }_{i}>0.6 \\ {\theta }_{j}>0.5\end{array}\right.$$

**Fig. 8:**
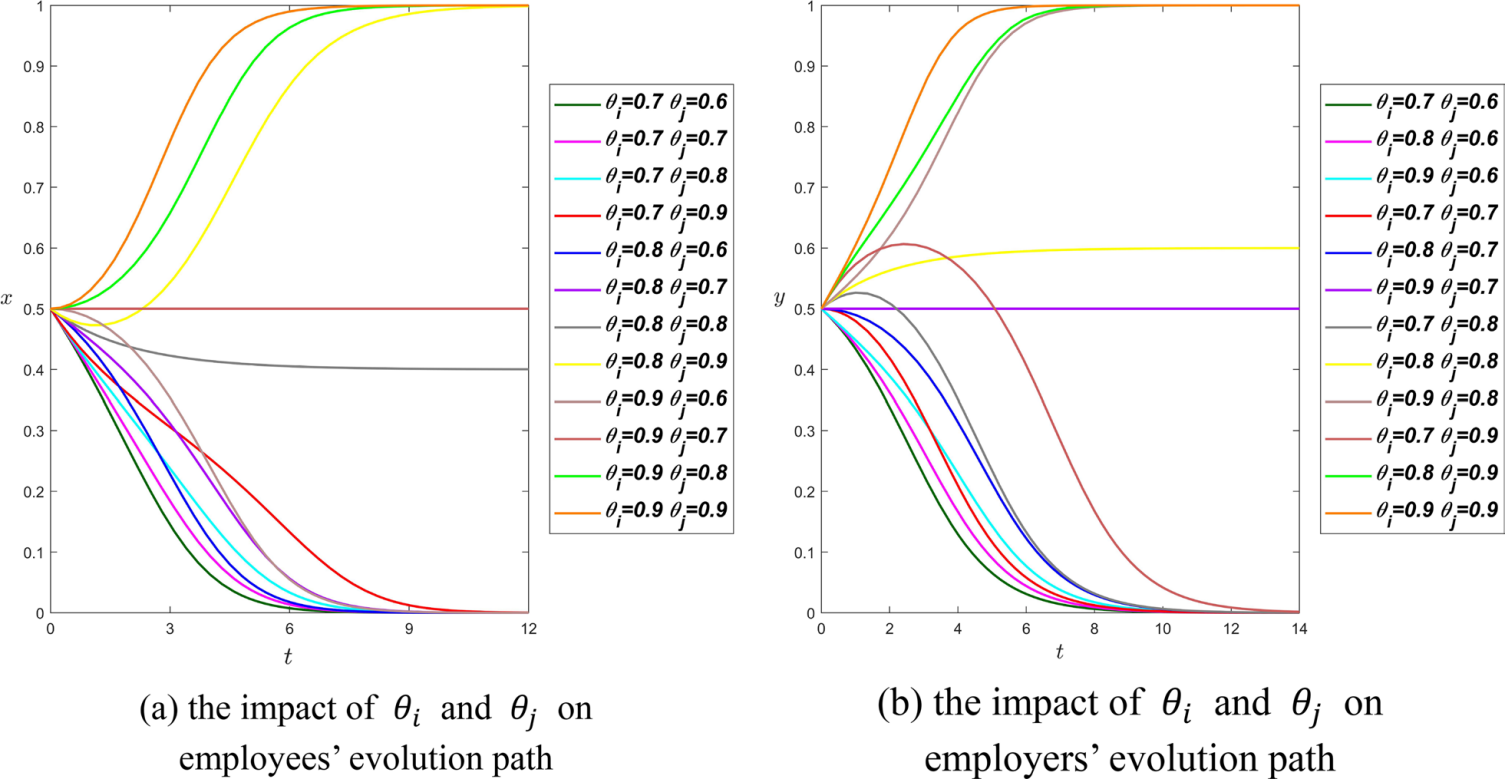
The influence curve of trust coefficient on evolution results under scenario 2

In scenario 2, the evolution rules of curves in figure (a) and (b) can be summarized as follows: in the lower part of figures, both parties tend to the equilibrium point $(\mathrm{0,0})$, that is, employees choose the strategy of involuntary overtime work, while employers choose the strategy of no overtime pay, in this case, the decrease of ${\theta }_{i}$ and ${\theta }_{j}$ can help shorten the evolution time of both parties. In the upper part of graphs, there are three curves in both graphs that tend to the point $(\mathrm{1,1})$, i.e., employees choose the strategy of voluntary overtime and employers choose the strategy of overtime payment. As ${\theta }_{i}$ and ${\theta }_{j}$ increase, the evolution curves all move closer to the $y$-axis. In addition, there are two abnormal curves in both graphs, ${\theta }_{i}=0.8,{\theta }_{j}=0.8$ and ${\theta }_{i}=0.9,{\theta }_{j}=0.7$. ${\theta }_{i}=0.8,{\theta }_{j}=0.8$ finally tends to $(\mathrm{0,0})$ around $t=85$, and ${\theta }_{i}=0.9,{\theta }_{j}=0.7$ tends to $(\mathrm{1,1})$ around $t=95$. In particular, when ${\theta }_{i}=0.9,{\theta }_{j}=0.9$, which satisfies ${\theta }_{i}{R}_{i}-{\beta }_{i}{R}_{j}>{2\alpha }_{i}{R}_{i}$ and ${\theta }_{j}{R}_{j}-{\beta }_{j}{R}_{i}>{2\alpha }_{j}{R}_{j}$, the system stabilizes at (Voluntarily, Pay) from Fig. 8, thus, Proposition 2 and Proposition 3 are confirmed here.

In either case, the trust coefficient has a consistent effect on both employees and employers. The decrease of ${\theta }_{i}$ and ${\theta }_{j}$ can help shorten the time for both parties to evolve to the equilibrium point (0,0), while the increase of ${\theta }_{i}$ and ${\theta }_{j}$ can accelerate the evolution of both parties to the equilibrium point of (1,1).

### Moral hazard

**Fig. 9:**
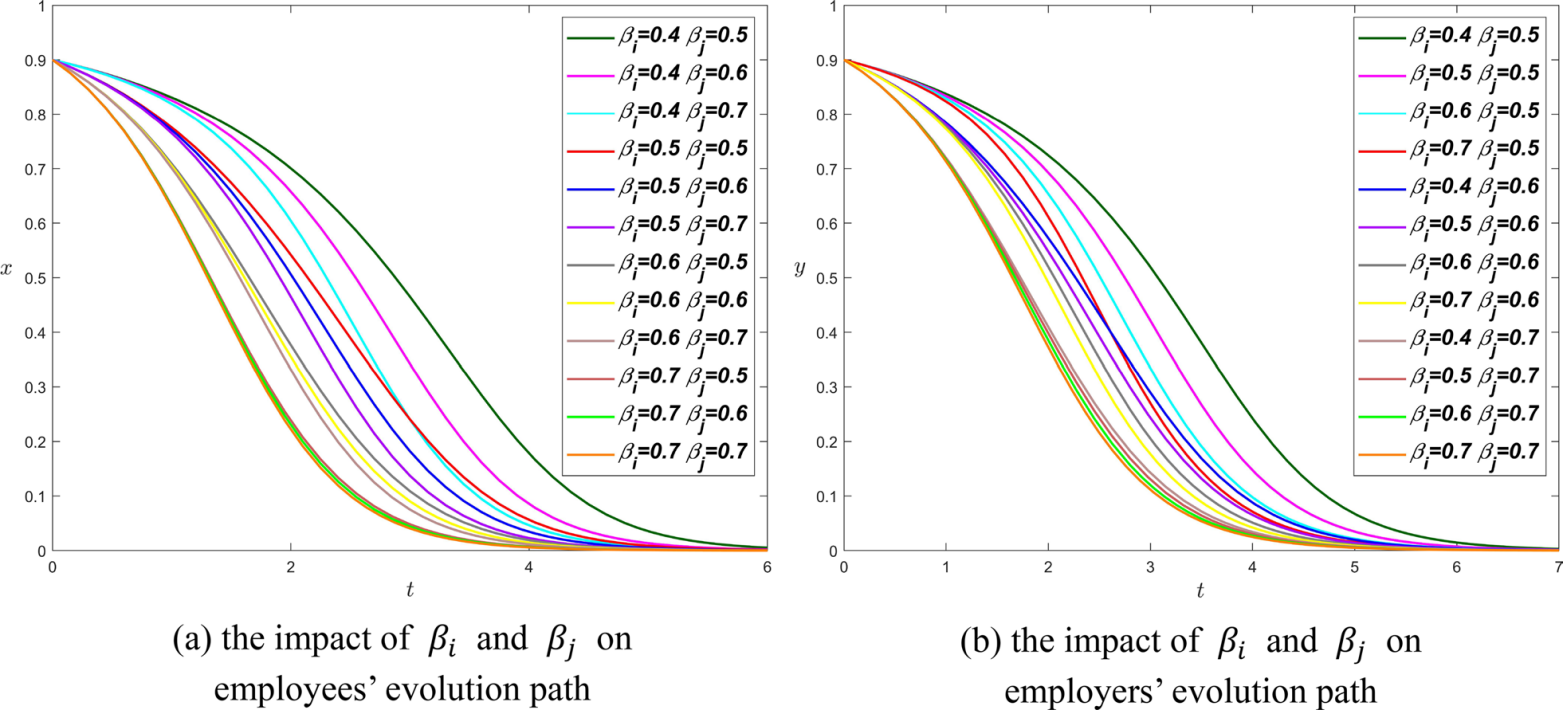
The influence curve of moral hazard on evolution results under scenario 1

**Scenario 1.** We set ${R}_{i}={R}_{j}=4$, ${\theta }_{i}={\theta }_{j}=0.8$, ${\alpha }_{i}=0.5$, and ${\alpha }_{j}=0.4$, thus

$$\left\{\begin{array}{c}0<0.8\times 4-{\beta }_{i}\times 4<0.5\times 4\\ 0<0.8\times 4-{\beta }_{i}\times 4<0.4\times 4\end{array}\right.\Rightarrow \left\{\begin{array}{c}0.3<{\beta }_{i}<0.8\\ 0.4<{\beta }_{j}<0.8\end{array}\right.$$

whereupon, we set ${\beta }_{i}=\mathrm{0.4,0.5,0.6,0.7}$ and ${\beta }_{j}=\mathrm{0.5,0.6,0.7}$. Figure 9 respectively shows the influence of the moral hazard coefficient on employees and employers. The patterns of these two graphs are very similar. As ${\beta }_{i}$ and ${\beta }_{j}$ increase, they get closer and closer to the $y$-axis. In other words, the higher the value of ${\beta }_{i}$ or ${\beta }_{j}$ is, the more stable the system is. The Proposition 1 is also confirmed from Fig. 9.

**Fig. 10:**
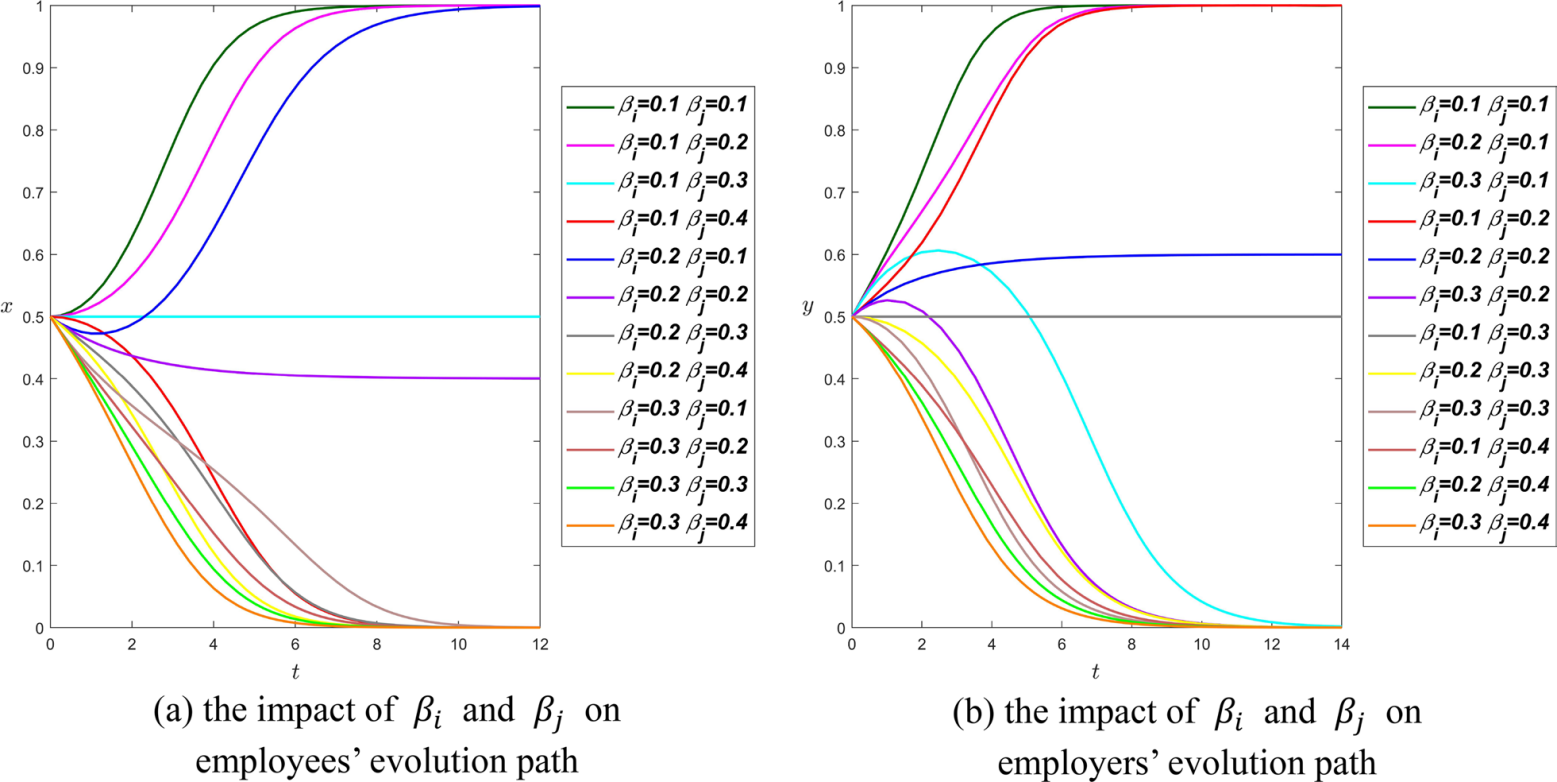
The influence curve of moral hazard on evolution results under scenario 2

**Scenario 2.** We set ${R}_{i}={R}_{j}=4$, ${\theta }_{i}={\theta }_{j}=0.7$, ${\alpha }_{i}=0.3$, and ${\alpha }_{j}=0.2$, thus

$$\left\{\begin{array}{c}0.7\times 4-{\beta }_{i}\times 4>0.3\times 4\\ 0.7\times 4-{\beta }_{i}\times 4>0.2\times 4\end{array}\right.\Rightarrow \left\{\begin{array}{c}{\beta }_{i}<0.4\\ {\beta }_{j}<0.5\end{array}\right.$$

we set ${\beta }_{i}=\mathrm{0.1,0.2,0.3}$ and ${\beta }_{j}=\mathrm{0.1,0.2,0.3,0.4}$. There are three lines ${\beta }_{i}=0.1,{\beta }_{j}=0.1$, ${\beta }_{i}=0.2,{\beta }_{j}=0.1$ and ${\beta }_{i}=0.1,{\beta }_{j}=0.2$ in the upper part of the two graphs in Fig. 10, all three lines have low moral hazard values. This means that when the moral hazard coefficients are small, both parties will reach equilibrium at point $(\mathrm{1,1})$, and reducing the values of ${\beta }_{i}$ and ${\beta }_{j}$ will help shorten the time to reach equilibrium. As the moral hazard coefficients increase, the equilibrium point will change from $(\mathrm{1,1})$ to $(\mathrm{0,0})$. The larger ${\beta }_{i}$ and ${\beta }_{j}$ are, the shorter the time for both parties to reach the point $(\mathrm{0,0})$. In addition, there are two other curves, ${\beta }_{i}=0.1,{\beta }_{j}=0.3$ and ${\beta }_{i}=0.2,{\beta }_{j}=0.2$, which both end up at $(\mathrm{0,0})$ around $t=84$. The Proposition 2 is also confirmed from Fig. 10.

In summary, as ${\beta }_{i}$ and ${\beta }_{j}$ increase, the equilibrium goes from $(\mathrm{1,1})$ to $(\mathrm{0,0})$. In practice, we hope that employees and employers reach the equilibrium point $(\mathrm{1,1})$, that is, employees choose the strategy of voluntary overtime work, and employers to choose the strategy of providing overtime pay. Therefore, the moral hazard coefficient of both sides should be reduced as much as possible.

## Discussion

### Research results

Based on the principal-agent theory, this study applies the evolutionary game method to construct a $2\times 2$ asymmetric game matrix about the employee-employer relationship of overtime behavior from the perspective of information asymmetry. Through the above theoretical analysis and simulation experiments, the main findings are as follows:

1. In any case, the system has five equilibrium points, an ESS point, and a saddle point. There are two scenarios depending on the constraints of the four parameters: resource consumption, information asymmetry coefficient, trust coefficient, and moral hazard coefficient. Based on the size of the determinant and trace at five equilibrium points, we put forward two theorems and three propositions, which are verified not only theoretically but also by data simulation.
2. The strategies of the employees and the employers will evolve from the initial state to (Involuntarily, Not pay) or (Voluntarily, Pay) under different situations. This is closely related to the initial parameters of the evolutionary game model and the payment matrix. However, (Voluntarily, Pay) is the Pareto optimal state, while (Involuntarily, Not pay) does not conform to social expectations and social morality. Employers' behavior of not providing overtime payment violates social ethics and relevant laws and regulations. Such moral anomie will hinder the normal operation of society and the development of organizations.
3. By summarizing the influence of each parameter on the evolution path, we can notice that fairness and information equivalence between employees and employers can effectively promote both parties to reach the Pareto optimal state. For example, in Fig. 4, a special curve will appear if both parties invest the same number of resources, which will take a long time to reach equilibrium. However, if the resource gap between the two parties is large, it can only reach a stable state (Involuntarily, Not pay) at last. Only when the number of resources invested by both parties is similar but not equal, can the optimal stable state (Voluntarily, Pay) be achieved. In Fig. 6, the optimal stable state (Voluntarily, Pay) can be achieved only if both parties have a small information asymmetry coefficient. In other words, employees and employers need to communicate and share information promptly to ensure the unity of information acquired by each other, thus achieving a win–win situation.
4. In order to facilitate the evolution of employees and employers from a stable state (Involuntarily, Not pay) to an optimal stable state (Voluntarily, Pay), four parameters should satisfy the following conditions: (a) Employees and employers should devote similar but not equal resources to overtime work. (b) Reduce information asymmetry between employees and employers to ensure that both sides have equal information. (c) Raise the level of trust between employees and employers, and trust can help promote a win–win optimal situation. (d) Reduce moral hazard between employees and employers.

### Management implications

The findings of this paper are helpful to understand the evolutionary logic of overtime behavior in organizations, which has good theoretical significance and practical value.

1. In the practice of working overtime in organizations, if employees choose to work overtime involuntarily, while employers do not provide overtime pay, such a strategy of evolutionary stability is against social morality and relevant laws, which not only damages the physical and mental health of employees but also harms the reputation and honor of the organization. In this case, it is necessary to introduce third-party supervision to strengthen the supervision of unpaid overtime work and reduce a series of moral hazard problems caused by overtime. Therefore, the government should regulate the rights and interests of employees by the law more strictly, improve the trade union system in enterprises more widely, thus establish a reasonable labor supervision system. Meanwhile, organizations must take social responsibility and stop exchanging employees' well-being for surplus value.
2. If employees choose to work overtime voluntarily and employers are willing to provide overtime payment, the system will dynamically converge to the Pareto optimal equilibrium point after the joint efforts of employers and employees in the long run. This is beneficial and sustainable for the development of the organization as well as the growth of employees. The organization's attitude towards overtime should adapt to the increasing needs of the new era, the need for sustainable social development, and the need for economic development to maintain progress. Even if employees choose to work overtime voluntarily, organizations should also minimize unnecessary overtime, which can be reduced by adopting new technologies and optimizing management processes to improve work efficiency.
3. Reduce information asymmetry between employees and the organization. First of all, the organization should help employees to establish a sense of "ownership", and share the success and benefits of the enterprise with them through the implementation of an employee stock ownership plan, which is bound to greatly improve the enthusiasm of employees. Second, pay attention to the compensation effect of overtime pay and further provide a high level of remuneration that can satisfy employees. The form of overtime pay is not limited to material payment, but can also recognize their efforts and achievements through the promotion of positions and awarding of personal honors, so as to realize the sense of accomplishment and life value of employees. Third, the assignment of work tasks should take urgency and task quantity into consideration, maintain the balance of task quantity as well as promote work-family balance among employees.

### Strengths, limitations, and future research

This study innovatively applies the evolutionary game method to solve the problem in organizational behavior, that is, the ubiquitous overtime problem. Previous studies on overtime work mainly use multi-layer linear model and multiple regression model, but these two methods not only fail to show the dynamic evolution process between employees and employers, but also cannot explain the information asymmetry between them. However, we believe that the evolutionary game model is closer to the actual situation, and there is indeed a game between employees and employers about overtime. Moreover, the evolutionary game model can well reflect the information asymmetry between the two sides. Finally, our research complements the literature on overtime and principal-agent theory.

There are several limitations. First, this paper uses simulation experiments to verify the model, while actual case data may help to better understand the game between employees and employers. Second, in addition to information asymmetry and overtime pay, there may be other factors that influence whether employees volunteer to work overtime. Third, as we mentioned in the findings, third-party supervision needs to be introduced, so the three-way game between employees, employers, and the government may better interpret the mechanism of overtime.

Future research can consider the three-way game among employees, employers, and the government. Besides information asymmetry and overtime payment, other factors need to be taken into account. In addition, if possible, subsequent studies can validate the model with actual case data.

## Conclusion

This research shows that the strategic choice of employees and employers will eventually be in (Involuntarily, Not pay) or Pareto optimal state (Voluntarily, Pay), which is closely related to resource consumption, information asymmetry coefficient, trust coefficient, and moral hazard coefficient. By summarizing the influence of each parameter on the evolutionary path of employees and employers, it can be found that fairness and information equivalence between employees and employers can effectively promote both parties to reach Pareto optimal state. In other words, employees and employers need to communicate and share information promptly to ensure that the information obtained by each other is consistent. The findings of this paper are helpful to understand the evolutionary logic of organizational overtime behavior, provide theoretical guidance for scientific management of employees' overtime behavior, and further improve employees' working psychology.

## Data Availability

Data sharing is not applicable to this article as no datasets were generated or analyzed during the current study.

## Abbreviations

ESS: Evolutionary stability strategy.
RD: Replication dynamics
det: Determinant.
tr: Trace

## References

[1] Wang JJ (2020). How managers use culture and controls to impose a ‘996’work regime in China that constitutes modern slavery. Accounting Finance.

[2] Hochschild A, Machung A (2012). The second shift: Working families and the revolution at home.

[3] Dembe AE, Erickson JB, Delbos RG, Banks SM (2005). The impact of overtime and long work hours on occupational injuries and illnesses: new evidence from the United States. Occup Environ Med.

[4] Major VS, Klein KJ, Ehrhart MG (2002). Work time, work interference with family, and psychological distress. J Appl Psychol.

[5] Friedman D (1991). Evolutionary games in economics. Econom J Econom Soc.

[6] Cheng L, Liu G, Huang H, Wang X, Chen Y, Zhang J (2020). Equilibrium analysis of general N-population multi-strategy games for generation-side long-term bidding: an evolutionary game perspective. J Clean Prod.

[7] Chen H, Chen H-T (2021). The role of social network sites on the relationship between game users and developers: an evolutionary game analysis of virtual goods. Inf Technol Manag.

[8] Wang L, Schuetz CG, Cai D (2021). Choosing response strategies in social media crisis communication: an evolutionary game theory perspective. Inf Manag.

[9] Yang H, Hu Y, Qiao H, Wang S, Jiang F (2020). Conflicts between business and government in bike sharing system. Int J Confl Manag.

[10] Watanabe M, Yamauchi K (2016). Psychosocial factors of overtime work in relation to work-nonwork balance: a multilevel structural equation modeling analysis of nurses working in hospitals. Int J Behav Med.

[11] Nijp HH, Beckers DGJ, Geurts SAE, Tucker P, Kompier MAJ (2012). Systematic review on the association between employee worktime control and work-non-work balance, health and well-being, and job-related outcomes. Scand J Work Environ Health.

[12] Min A, Hong HC, Son S, Lee T (2021). Sleep, fatigue and alertness during working hours among rotating-shift nurses in Korea: an observational study. J Nurs Manag.

[13] Beldon R, Garside J (2022). Burnout in frontline ambulance staff. J Paramedic Pract.

[14] Peterson SA, Wolkow AP, Lockley SW, Brien CS, Qadri S, Sullivan JP (2019). Associations between shift work characteristics, shift work schedules, sleep and burnout in North American police officers: a cross-sectional study. BMJ Open.

[15] Beckers DGJ, van der Linden D, Smulders PGW, Kompier MAJ, Taris TW, Geurts SAE (2008). Voluntary or involuntary? Control over overtime and rewards for overtime in relation to fatigue and work satisfaction. Work Stress.

[16] Golden L (2009). A brief history of long work time and the contemporary sources of overwork. J Bus Ethics.

[17] Liu B, Chen H, Yang X, Hou C (2019). Why work overtime? A systematic review on the evolutionary trend and influencing factors of work hours in China. Front Public Health.

[18] Van der Hulst M (2003). Long workhours and health. Scand J Work Environ Health.

[19] Schein EH (2010). Organizational culture and leadership.

[20] Premack SL, Wanous JP (1985). A meta-analysis of realistic job preview experiments. J Appl Psychol.

[21] Feldman DC (2002). Managers' propensity to work longer hours: a multilevel analysis. Hum Resour Manag Rev.

[22] Perlow LA (1999). The time famine: toward a sociology of work time. Adm Sci Q.

[23] Laurence GA, Fried Y, Raub S (2016). Evidence for the need to distinguish between self-initiated and organizationally imposed overload in studies of work stress. Work Stress.

[24] Deci EL, Ryan RM (2000). The" what" and" why" of goal pursuits: Human needs and the self-determination of behavior. Psychol Inq.

[25] Michelacci C, Pijoan-Mas J. The effects of labor market conditions on working time: the US-EU experience. 2007.

[26] Barrick MR, Mount MK (1991). The big five personality dimensions and job performance: a meta-analysis. Pers Psychol.

[27] Van der Hulst M, Geurts S (2001). Associations between overtime and psychological health in high and low reward jobs. Work Stress.

[28] Beckers DG, van der Linden D, Smulders PG, Kompier MA, Taris TW, Van Yperen NW (2007). Distinguishing between overtime work and long workhours among full-time and part-time workers. Scand J Work Environ Health.

[29] Hobfoll SE (1989). Conservation of resources: a new attempt at conceptualizing stress. Am Psychol.

[30] Akerlof GA, Diamond P, Rothschild M (1978). The market for “lemons”: quality uncertainty and the market mechanism. Uncertainty in economics.

[31] Bergh DD, Ketchen DJ, Orlandi I, Heugens PPMAR, Boyd BK (2019). Information asymmetry in management research: past accomplishments and future opportunities. J Manag.

[32] Naqvi SK, Shahzad F, Rehman IU, Qureshi F, Laique U (2021). Corporate social responsibility performance and information asymmetry: the moderating role of analyst coverage. Corp Soc Responsib Environ Manag.

[33] Wang R (2021). Information asymmetry and the inefficiency of informal ip strategies within employment relationships. Technol Forecast Soc Chang.

[34] Wang S (2021). Seeing differently from others: the impact of relationship conflict asymmetry and realization on team performance. Int J Confl Manag.

[35] Andrade-Rojas MG, Kathuria A, Konsynski BR (2021). Competitive brokerage: how information management capability and collaboration networks act as substitutes. J Manag Inform Syst.

[36] Sousa-Poza A, Ziegler A (2003). Asymmetric information about workers' productivity as a cause for inefficient long working hours. Labour Econ.

[37] Golden L. Distinctions between overemployment, overwork, workaholism and heavy investments in work time. In: Harpaz and Snir book, Heavy work investment. 2014. doi: 10.2139/ssrn.2371082.

[38] Smit BW, Montag-Smit T (2018). The role of pay secrecy policies and employee secrecy preferences in shaping job attitudes. Hum Resour Manag J.

[39] DeRiviere L (2008). Lower monetary returns for that many overtime hours? Forget it!. J Socio-Econ.

[40] Bratti M, Staffolani S (2007). Effort-based career opportunities and working time. Int J Manpow.

[41] Mazzetti G, Schaufeli WB, Guglielmi D, Depolo M (2016). Overwork climate scale: psychometric properties and relationships with working hard. J Manag Psychol.

[42] Huang HW, Xia X, Zhao WX, Pan XQ, Zhou XQ (2020). Overwork, job embeddedness and turnover intention among Chinese knowledge workers. Asia Pac J Hum Resour.

[43] Roca CP, Cuesta JA, Sánchez A (2009). Evolutionary game theory: temporal and spatial effects beyond replicator dynamics. Phys Life Rev.

[44] Liu J, Yu C, Li C, Han J (2020). Cooperation or conflict in doctor-patient relationship? An analysis from the perspective of evolutionary game. IEEE Access.

[45] Wang G, Chao Y, Cao Y, Jiang T, Han W, Chen Z (2022). A comprehensive review of research works based on evolutionary game theory for sustainable energy development. Energy Rep.

[46] Taylor PD, Jonker LB (1978). Evolutionary stable strategies and game dynamics. Math Biosci.

[47] Yan H, Wei H, Wei M (2021). Exploring tourism recovery in the post-COVID-19 period: an evolutionary game theory approach. Sustainability.

[48] Cressman R (1992). The stability concept of evolutionary game theory: a dynamic approach.

[49] Cressman R (1995). Evolutionary game theory with two groups of individuals. Games Econom Behav.

[50] Schuster P, Sigmund K, Hofbauer J, Gottlieb R, Merz P (1981). Selfregulation of behaviour in animal societies. Biol Cybern.

[51] Miller GJ (1992). Managerial dilemmas: the political economy of hierarchy.

[52] Holmstrom B, Milgrom P (1987). Aggregation and linearity in the provision of intertemporal incentives. Econometrica.

[53] Rowell D, Connelly LB (2012). A history of the term “moral hazard”. J Risk Insur.

[54] Güth W, Klose W, Königstein M, Schwalbach J (1998). An experimental study of a dynamic principal–agent relationship. Manag Decis Econ.

